# Successful clearance of persistent *Staphylococcus aureus* pneumonia with high-dose continuous infusion cefazolin

**DOI:** 10.1128/asmcr.00114-24

**Published:** 2025-03-31

**Authors:** Annie A. Smelter, Christopher R. Frei, Dan F. Smelter

**Affiliations:** 1 Joe R. and Teresa Lozano Long School of Medicine, The University of Texas Health at San Antonio 12345, San Antonio, Texas, USA; 2 College of Pharmacy, The University of Texas at Austin 15528, Austin, Texas, USA; Pattern Bioscience, Austin, Texas, USA

**Keywords:** beta-lactams, continuous infusion, CABP, MSSA

## Abstract

**Background:**

Deep-seated *Staphylococcus aureus* infections are difficult to treat and require appropriate antibiotic regimens for clinical success. Recent studies have shown that continuous infusion (CI) of β-lactam antibiotics is associated with reduced mortality, though optimal dosages remain to be determined. Here, we present a case in which high-dose cefazolin, at 10 g daily CI, was used to safely and successfully clear methicillin-susceptible *S. aureus* (MSSA) pneumonia.

**Case Summary:**

A 33-year-old male was admitted to the medical intensive care unit with suspected MSSA community-acquired pneumonia. Over the course of a 43-day hospital stay, the patient’s pneumonia relapsed twice following two 5- to 7-day courses of cefazolin 8 g daily CI. Clearance of the pneumonia was achieved after a 14-day course of cefazolin 10 g daily CI, and the patient was discharged.

**Conclusion:**

Here, we describe a patient with incomplete clearance of MSSA pneumonia despite two 5- to 7-day courses of cefazolin 8 g daily CI. Following a 14-day course of cefazolin 10 g daily CI, the patient’s fever and leukocytosis rapidly resolved without direct evidence of drug-related toxicity. This case report provides evidence for the safe and effective use of high-dose cefazolin CI in the clearance of persistent MSSA pneumonia.

## INTRODUCTION

Methicillin-susceptible *Staphylococcus aureus* (MSSA) pneumonia often manifests as a deep-seated infection that is notoriously challenging to treat, requires a prolonged treatment duration, and is associated with higher rates of treatment failure.

Cefazolin, a first-generation cephalosporin, has been shown to be safe and effective in treating many bacterial infections, including MSSA pneumonia. The standard recommended cefazolin dosing for this indication is 2 g administered intravenously every 8 h (q8h), totaling 6 g daily, for a minimum duration of 5 to 7 days per the Infectious Diseases Society of America and American Thoracic Society guidelines (1).

Recent high-profile studies evaluating the traditional dosing canon for β-lactams indicate that prolonged or continuous infusion (CI) administration may produce similar outcomes compared to the standard intermittent dosing, despite not being routinely performed (2–4). Under certain disease states, it may be more beneficial to administer β-lactams via CI as opposed to traditional intermittent infusion, but the published data is insufficient.

Furthermore, while the ANCEF (brand name cefazolin for injection) package insert states that “in rare instances, doses up to 12 g of cefazolin per day have been used,” there is a paucity of data detailing the safety and efficacy of high-dose cefazolin (i.e., 10+ grams/day). Several retrospective studies have reported dosing of cefazolin up to 12 g daily, but none report greater than 8 g daily for the entire treatment duration (5–7). To our knowledge, this is the first report to show 10 g daily CI for 2 weeks, with nearly 4 weeks total of cefazolin therapy at ≥8 g daily, without any indication of adverse events or unsafe findings. Here, we present a case in which high-dose cefazolin, at 10 g daily CI, was used to safely and effectively clear persistent MSSA pneumonia.

## CASE PRESENTATION

**Fig 1:**
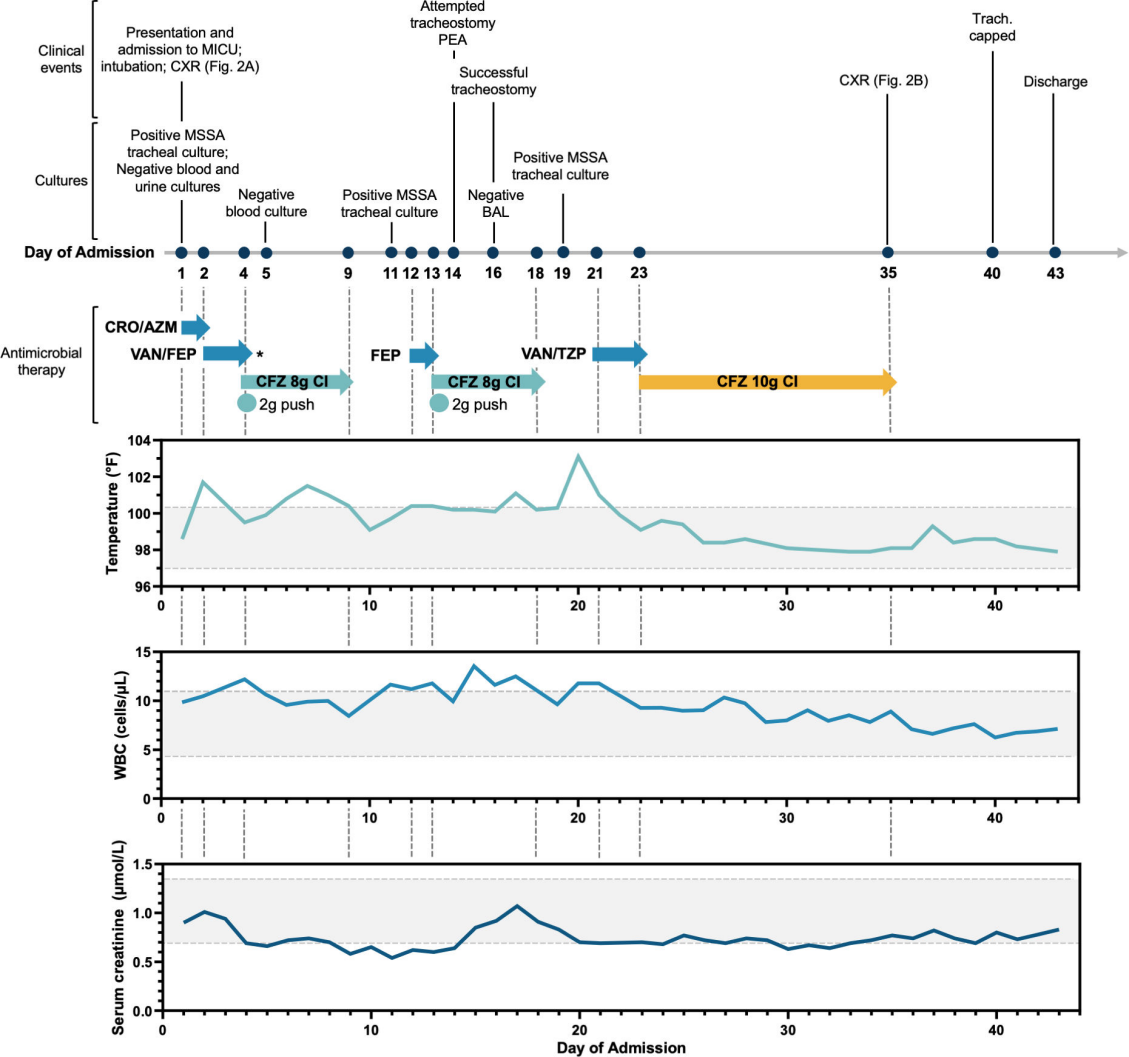
Summary of hospital course. Timeline detailing clinical events, cultures, and antimicrobial therapy throughout the 43-day hospital stay with temperature, white blood cell (WBC) count, and serum creatinine with normal value range shaded. Serum creatinine is included as an analog for renal function. CXR, chest X-ray; CI, continuous infusion; CRO, ceftriaxone (1 g q24h push); AZM, azithromycin (500 mg q24h); FEP, cefepime (2 g q8h push); TZP, piperacillin/tazobactam (4.5 g q8h over 4 h); VAN, vancomycin; CFZ, cefazolin. IV push administered over 2 to 5 min. *Note: Last VAN dose 8 h after CFZ start on D4.

**Fig 2:**
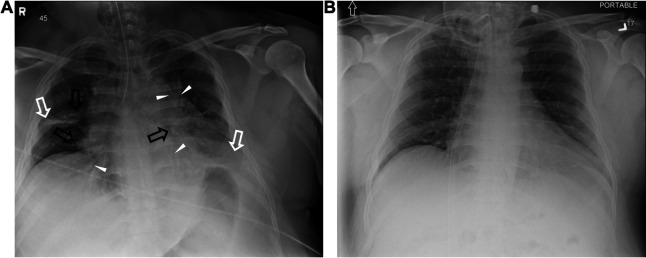
Chest X-rays from admission and post-antimicrobial therapy. (**A**) AP CXR showing enlarged cardiomediastinal silhouette with low lung volumes, associated right middle lobe, and bilateral infrahilar opacities (black arrows) with air bronchograms (white triangles), fluid in minor fissure, and likely left pleural effusion (white arrows) (day of admission). (**B**) AP CXR showing improved aeration of bilateral lungs and interval resolution of pulmonary edema and parenchymal opacities, without areas of focal consolidation (day 35 of hospitalization).

A 33-year-old male with a past medical history significant for obesity, sleep apnea, methamphetamine use, and a recent tooth infection presented to the emergency department (ED) via emergency medical services at 4:00 a.m. for multiple episodes of severe apnea after a family member found the patient unresponsive in his sleep (day 1 of admission, Fig. 1). Initial vital signs were remarkable for tachypnea, tachycardia at 119 beats per minute, PaO_2_ saturation of 57%, blood pressure of 96/58 mmHg, body mass index of 37.5 kg/m^2^, and temperature 98.6°F. Upon presentation to the ED, he appeared somnolent and not oriented with appreciable fixed, constricted pupils. Following two rounds of 0.4 mg naloxone and sternal rub, he showed modest improvement and dilation of pupils; however, clinical lab analysis of a urine sample later identified only amphetamine metabolites. Lungs were clear to auscultation bilaterally, and the patient denied any chest pain or shortness of breath. Although the patient had recently been diagnosed with sleep apnea, he reported not tolerating CPAP use at home. The patient initially responded well to CPAP/BiPAP with PaO_2_ reaching normal levels but then demonstrated a decline in mental status until he became unresponsive with PaO_2_ desaturation down to 60% and suspected hypoxic seizure, requiring intubation. Chest X-ray (CXR) at presentation demonstrated low lung volumes, right middle lobe and bilateral infrahilar opacities, and air bronchograms concerning for pneumonia with likely left pleural effusion (see Fig. 2A). The patient was started on empiric ceftriaxone (1 g q24h) and azithromycin (500 mg q24h) for community-acquired bacterial pneumonia coverage and admitted to the medical intensive care unit (MICU). Blood and urine cultures collected at presentation were negative for growth. At the time of intubation, tracheal cultures were positive for MSSA with oxacillin susceptibility ≤0.25 mg/L.

Notable details for the 43-day hospital course and pertinent laboratory values are provided in Fig. 1. Briefly, the patient was sedated and appeared stable for the rest of the first day of admission (D1), but overnight into D2 became febrile (101.7°F) and hypotensive (94/45 mmHg), requiring pressors. Pulmonary exam revealed coarse breath sounds. Antimicrobial therapy was changed to vancomycin (2 g loading dose and 1 g q8h) and cefepime (2 g q8h) on D2 for ventilator-associated pneumonia (VAP) coverage. Vancomycin dosing was determined using the Cockroft-Gault equation to estimate glomerular filtration rate using serum creatinine. Over the next 3 days, the medical team ruled out aspiration pneumonia and, with pharmacy consult, determined that the vancomycin regimen produced subtherapeutic levels even with daily increases in dosing (highest measured trough concentration was 8.5 mg/L, with a goal of 10–20mg/L). On D4, the antimicrobial regimen was re-evaluated following the reassessment of initial indications of pneumonia; cefepime was discontinued and replaced with cefazolin 8 g daily CI (with one cefazolin 2 g loading dose given as IV push over 2 to 5 min and one vancomycin 1.5 g dose). D5 blood cultures continued to be negative.

On D9, cefazolin was discontinued after a 7-day antimicrobial course (5 days cefazolin 8 g daily CI), despite temperature remaining elevated (100.4°F). The medical team noted “no sign of active infection” on D10 and marked “leukocytosis” and “septic shock” as resolved in notes. The patient’s white blood cell (WBC) count became elevated again on D11 and by D12 was febrile (100.4°F) and required aggressive suction for pulmonary secretions. Cefepime (2 g q8h) therapy was initiated empirically and on D13 was changed to cefazolin 8 g daily CI. As long-term intubation was expected, a tracheostomy was attempted on D14. However, copious secretions and mucous plugs obscured the view, and the patient suffered a brief cardiopulmonary arrest. A successful tracheostomy was performed on D16, and a bronchoalveolar lavage returned negative for pathogenic microorganisms (10,000 CFU/mL usual respiratory flora, 1+ epithelial cells, 1+ WBCs). The patient’s fever and leukocytosis persisted through D18 when cefazolin treatment was discontinued at the completion of the second set of 7 days of antimicrobial therapy. By D20, the patient reached a high fever (103.1°F) and was noted to have thick airway secretions. Vancomycin (2.5 g loading dose, then 1 g q6h) and piperacillin/tazobactam (4.5 g q8h) were initiated for VAP coverage on D21. When respiratory cultures identified MSSA, antimicrobial therapy was changed to high-dose cefazolin 10 g daily CI for 14 days on D23. The patient’s fever and leukocytosis improved steadily, with persistent thick oral and tracheal secretions for the first week that improved during the second week of cefazolin 10 g daily CI. Serum creatinine remained within the normal range or slightly below (0.7–1.3 mg/dL reference range) throughout the duration of high-dose cefazolin therapy. CXR on D35 showed improvement in aeration and resolution of pulmonary edema, parenchymal opacities, and areas of consolidation (see Fig. 2B). The patient’s condition improved such that his tracheostomy tube was capped on D40, and he was stable on room air until discharge to home care on D43.

## DISCUSSION

Here, we describe a patient with MSSA pneumonia of unclear etiology with incomplete clearance after two week-long courses of cefazolin 8 g daily CI. Success was achieved following a 14-day course of cefazolin 10 g daily CI: the patient’s condition improved with rapid and lasting resolution of fever and leukocytosis without evidence of toxicity or adverse effects, providing evidence for the use of both high-dose cefazolin and CI β-lactam therapy.

For more than five decades, cefazolin has been successfully used to treat bacterial pneumonias, particularly those caused by MSSA (8). However, deep-seated and complicated staphylococcal infections are still associated with high morbidity and mortality, having 30-day mortality rates near 30% (9, 10). Cefazolin is often the preferred antibiotic for treating infections caused by MSSA as it has a favorable dosing regimen and a robust safety profile compared to similar anti-staphylococcal penicillins (ASPs) such as nafcillin or oxacillin (11). Recent studies have found cefazolin to be associated with lower mortality rates, less nephrotoxicity, and reduced clinical failure or discontinuation due to adverse events when compared to ASPs (12–14).

One concern with cefazolin use is the potentially reduced penetrance into compartments of the body such as the cerebrospinal fluid when treating meningitis (5, 15) or, as in our patient’s case, penetrance into the lungs. To our knowledge, there is no available data on the penetration of cefazolin into the lung epithelial lining fluid. However, pleural fluid measurements of cefazolin indicate that standard intermittent dosing achieves concentrations sufficient for therapeutic efficacy (16). If penetration of the antibiotic is a concern, an increase in dosage will likely lead to increased drug concentration at the site of interest, including the lungs or cerebrospinal fluid, especially in the setting of inflammation. Furthermore, through increasing the antibiotic dose and/or dosing frequency, it is likely easier to achieve maximal time above the minimum inhibitory concentration (T > MIC), particularly for infection sources within highly vascularized tissues.

Recent major reports and a large clinical trial (BLING III) have focused on CI versus intermittent infusions of β-lactam antibiotics, and the data suggest that CI is associated with reduced mortality compared to intermittent infusion, likely due to maximizing T > MIC (3, 4, 17). β-lactam antibiotics are known for having short half-lives, which require frequent dosing to maintain serum concentrations over the bacterial MIC. This is something that can be overcome through the use of CI or prolonged dosing regimens.

Many institutions do not currently use CI dosing for antibiotics, and incorporating it as an institution policy likely faces challenges. Deciding when CI or intermittent infusion is appropriate for the patient requires further studies, particularly if CI allows for increased total daily drug dosage (such as 10+ grams/day cefazolin) without the risk associated with high peak serum concentrations that come with intermittent infusion. However, CI regimens increase the healthcare burden and workload for nursing and pharmacy staff, and the extended duration of infusion can introduce new risks such as drug stability.

This report demonstrates nearly 4 weeks total of high-dose cefazolin CI therapy without directly attributable toxicity due to the drug. Throughout the high-dose cefazolin therapy, the patient’s serum creatinine stayed within the normal range, and there was no indication of hypoprothrombinemia (INR never went above 1.6) (7, 18).

### Conclusion

High-dose cefazolin (10 g daily), given as a CI, was able to clear community-acquired MSSA pneumonia while not exhibiting any signs of toxicity or adverse effects, highlighting the wide therapeutic index of this first-generation cephalosporin. The recurrence of pneumonia twice throughout the patient’s hospital course, before receiving the higher dose of cefazolin CI, suggests incomplete clearance of the infection that may have been averted with a longer duration of therapy or higher initial dosing of cefazolin. Clinicians should consider consultation with the infectious diseases team when patients have complex and severe infections caused by *S. aureus*.

## Data Availability

Data are available upon request.
